# Zanamivir versus trivalent split virus influenza vaccine: a pilot randomized trial

**DOI:** 10.1111/irv.12301

**Published:** 2015-01-04

**Authors:** Brenda L Coleman, Shaza A Fadel, Steven J Drews, Todd F Hatchette, Allison J McGeer

**Affiliations:** a Dalla Lana School of Public Health, University of TorontoToronto, ON, Canada; b Department of Microbiology, Mount Sinai HospitalToronto, ON, Canada; c ProvLabCalgary, AB, Canada; d Microbiology, Immunology and Infectious Diseases, University of CalgaryCalgary, AB, Canada; e Department of Pathology, Dalhousie UniversityHalifax, NS, Canada; f Department of Pathology and Laboratory Medicine, Queen Elizabeth Health Sciences CentreHalifax, NS, Canada

**Keywords:** Adult, antiviral, influenza, respiratory tract infections

## Abstract

**Background:**

Healthcare workers may be exposed to people with respiratory viral infections more often than other working adults. Understanding the risk and the effectiveness of different preventive measures is of great importance.

**Objectives:**

To estimate adherence to prophylactic antiviral medication for a full influenza season, to the compare efficacy of antiviral prophylaxis to that of the seasonal influenza vaccine and to identify exposures that increase risk of acute respiratory illnesses (ARI) in healthy adults.

**Methods:**

Participants were randomized 1:2 to receive the 2008–2009 influenza vaccine or daily prophylaxis with 10 mg of zanamivir during the season. Web-based questionnaires collected information on demographics, symptoms, exposures, medication use and side effects.

**Results:**

Sixty-four healthy adults were recruited in November 2008. Three of 40 active participants discontinued zanamivir due to side effects; the remaining 37 took >85% of scheduled doses for a median of 121 days. Symptomatic, laboratory-confirmed influenza was detected in one person randomized to zanamivir (2·5%) and 2/20 (10%) who received the vaccine (*P* = 0·25). Forty-seven participants reported 109 episodes of ARI. Factors associated with an ARI were exposure to a spouse (OR 7·2), child (OR 2·4) or patient (OR 2·0) with symptoms of an ARI in the previous 7 days.

**Conclusions:**

Breakthrough influenza infection occurred in both vaccinated participants and those receiving antiviral prophylaxis. Most adults were willing and able to comply with season-long prophylaxis. Report of recent exposure to family members and patients with an ARI increased the risk of developing an ARI in healthy adults.

## Introduction

Respiratory tract infections are one of the most common causes of disease and in humans and for absenteeism in healthcare workers.^1^,^2^ In Canada, influenza-like illness consultation rates ranged from 15 to 20 per 1000 patients in the 2005–2006 and 2006–2007 influenza seasons, with 7–15% of illness caused by influenza.^3^,^4^ The primary tool for preventing influenza is annual vaccination. Osterholm *et al*.^5^ report that the efficacy of the trivalent inactivated influenza vaccines in eight randomized controlled trials of adults 18–65 years of age was 59%. Neuraminidase inhibitors may also be effective in preventing influenza, with estimated efficacy of 31–83% in placebo-controlled trials.^6^–^8^ Healthcare workers providing direct patient care may be at increased risk of developing influenza compared to other healthy adults^9^,^10^ and may transmit the disease to vulnerable patients making them a priority group for influenza vaccines and, in the event of a pandemic, for antiviral medication.^11^,^12^

During the 2008–2009 influenza season, this pilot study determined the adherence to antiviral medication for an influenza season, the relative efficacy of antiviral prophylaxis with zanamivir and seasonal influenza vaccine against laboratory-confirmed influenza infection, and determined risk factors for acute respiratory illnesses (ARI) among healthy adults.

## Methods

### Participants

Recruitment occurred between 5 November 2008 and 19 November 2008 at Mount Sinai Hospital, an acute care 472 bed academic health centre in Toronto, Canada. Participants were eligible if they were 18–69 years old, were available for follow-up during the study period and provided written informed consent. Individuals were excluded when they had any health or medical conditions that contraindicated influenza vaccine or zanamivir administration, had received the 2008–2009 influenza vaccine prior to the study start date, were pregnant or planning to be pregnant or were breastfeeding, had received immunoglobulin therapy within 6 months of study entry, were participating a study of any investigational drug during the study period or had an immunocompromising condition or therapy. Women of childbearing potential in the prophylaxis arm were required to have negative pregnancy tests prior to receiving each prescription for zanamivir.

### Study design

A non-blinded randomized controlled pilot study with participants randomized in a two to one ratio of antiviral prophylaxis to influenza vaccine. A random number table was used to generate assignments, in blocks of 4 or 6. A staff member not affiliated with the study generated the list and provided the assignment to study nurses following consent. Participants in the vaccine group received one intramuscular injection of Fluviral® (GlaxoSmithKline, Mississauga, ON, Canada) 2008–2009 trivalent inactivated split virion influenza vaccine containing influenza A/Brisbane/59/2007 (H1N1), A/Uruguay/716/2007 (H3N2) and B/Florida/4/2006. Participants in the antiviral prophylaxis arm were prescribed one 10 mg dose of zanamivir (Relenza®; GlaxoSmithKline) per day for the influenza season. Participants who experienced side effects and discontinued antiviral prophylaxis were given either Fluviral® or Vaxigrip® (Sanofi Pasteur, Toronto, ON, Canada) but remained in the antiviral arm of the study for analysis. The influenza season was defined, *a priori,* as starting when the proportion of all specimens tested for influenza in the province was >3% positive for 1 week or >2·5% for two consecutive weeks and ending when the proportion was less than 2% positive for two consecutive weeks. These definitions were established to keep the risk of exposure to influenza low for participants on prophylactic antiviral medication. This study was approved by Health Canada and Mount Sinai Hospital's Research Ethics Board, was registered with ClinicalTrials.gov (NCT00784784) and was conducted according to Good Clinical Practice guidelines.

### Data collection

**Figure 1:**
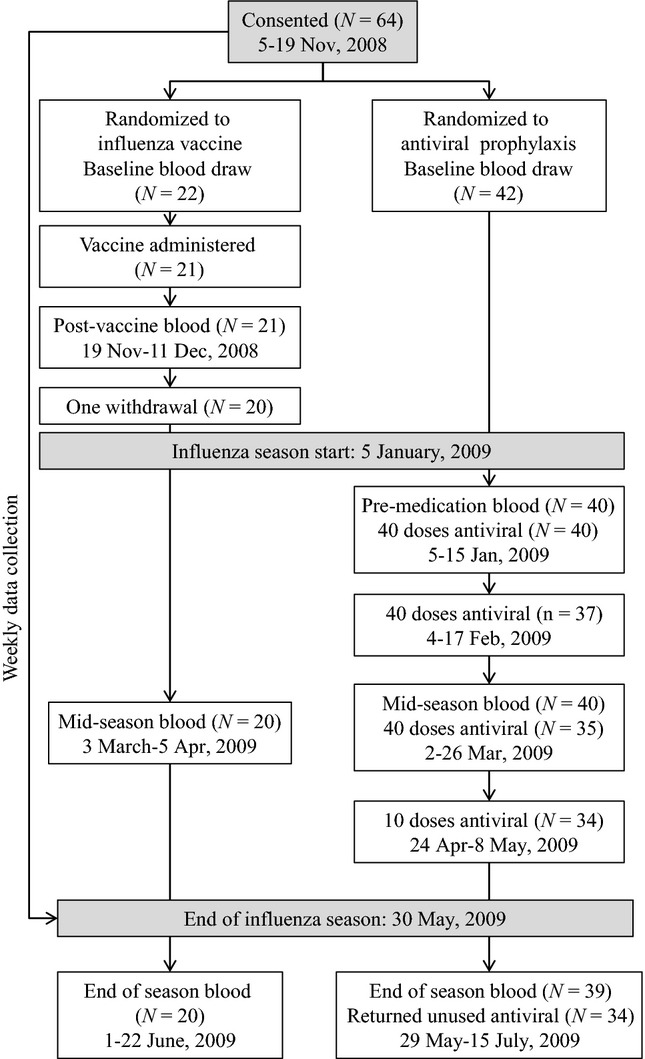
Flow chart of participation in clinical trial, November 2008 to June 2009.

Data collection started on the day of enrolment and continued until the end of the seasonal influenza season. Demographic, risk factor and health status information was collected by questionnaire at enrolment, 4 or 5 face-to-face interviews, weekly web-based diaries and daily illness questionnaires for all ARI. As shown in Figure1, blood samples were collected four times: at baseline (prior to vaccination for the vaccine group), on day 14 (+7) after vaccination (vaccine group) or at the start of influenza season (antiviral prophylaxis group), on day 24–38 of the influenza season (mid-season) and 2–3 weeks after the end of the influenza season. Sera were extracted and stored, frozen at −70°C for later testing. Active surveillance for acute respiratory illness occurred using web-based weekly diaries of symptoms throughout the influenza season. Participants with any acute respiratory symptom (runny or stuffy nose, sneezing, sore or scratchy throat, hoarseness, cough) or an unexplained fever were asked to have a nasopharyngeal (NP) swab collected by study nurses and to self-collected a mid-turbinate nasal swab.^13^ Adherence to prophylaxis was assessed using weekly diaries and medication counts at each of four study visits.

### Laboratory testing

All mid-turbinate nasal and NP swab samples were tested for influenza virus using the CDC influenza A matrix and influenza B NAAT at the Ontario Public Health Laboratory, Toronto, ON. Unused media was frozen at −70°C and tested using Seeplex® RV12 ACE (Seegene, Seoul, Korea) multiplex NAAT for 12 respiratory viruses: metapneumovirus, adenovirus A/B/C/D/E, human coronavirus 229E/NL63 and OC43, parainfluenza virus 1, 2 and 3, influenza A and B, respiratory synctial virus A and B and rhinovirus A/B at the end of the season.

Hemagglutination inhibition (HAI) assays were used to measure antibodies specific to influenza A/Brisbane/10/2007 (H3N2), A/Brisbane/59/2007 (H1N1), A/California/07/2009 (H1N1), A/Uruguay/716/2007 (H3N2), B/Florida/04/2006 (Yamagata) and B/Malaysia/2506/2004 (Victoria) in serum samples of each participant. HAIs were performed at Dalhousie University, Halifax, Nova Scotia using 0·5% turkey erythrocytes with 4 hemagglutinin units per 25 μl of virus based on the WHO-recommended procedure.^14^ Laboratory technicians were blinded to the arm to which the participants were assigned.

### Case definitions

Laboratory-confirmed influenza infection was defined as symptomatic infection, confirmed by NAAT testing of either NP or mid-turbinate nasal swabs. Seroconversion after vaccination was defined as post-vaccination titre ≥1:40 if the pre-vaccination titre was <1:10 or a fourfold or greater increase in HAI titres^15^. Acute respiratory illness was defined as a recent onset of at least two symptoms of which at least one was respiratory, among rhinorrhea/nasal congestion, sore throat, cough, headache or feverishness.^16^

### Analysis

All participants remained in the arm to which they were randomized for modified intention-to-treat analyses, with people who withdrew prior to the influenza season excluded.^17^ Conditional logistic regression, matched on week of illness onset, was used to account for time-varying risk factors such as exposure to ill family or patients. Robust variance estimates were used to adjust for correlation due to several ARI reports from the same participants. All weekly reports for people without signs or symptoms of a respiratory illness were used as controls. Weeks without a reported illness were not eligible for inclusion in the analyses. Assumptions of logistic regression modelling were checked using scatter plots of residuals against each predictor. All statistical analyses were performed using stata® v11 (StataCorp, College Station, TX, USA).^18^ A *P* value <0·05 was considered statistically significant and all tests were two-sided. A sensitivity analysis of the conditional logistic regression model was conducted in which people who discontinued antiviral prophylaxis were re-classified as being in the vaccine arm at 14-days postvaccination. As negligible differences in other covariate regression co-efficient estimates were detected (≤0·01), these results are not presented.

## Results

### Study participants

Of the 64 participants recruited into the study, 42 were randomized to the antiviral arm and 22 to the vaccine arm. As shown in Table1, baseline characteristics were similar between the two groups. The median age of the participants was 41 years, 69% were female and 21% had a chronic disease. All but three individuals (95%) worked at a health care facility and 41% provided direct patient care. Two individuals from the vaccine group and two from the prophylaxis group withdrew from the study prior to the start of the influenza season and are excluded from further analyses.

**Table 1:** Profile of study participants, Toronto, Canada, November 2008

	Vaccine (*n* = 22) Number (%)	Antiviral (*n* = 42) Number (%)
Age in years; Median (range)	42·5 (25–59)	41 (24–56)
Days in study; Median (range)	199·5 (0–207)	199 (0–207)
Female	17 (77·3%)	27 (64·3%)
One or more chronic diseases	4 (19·0%)	9 (21·4%)
Current smoker*	3 (14·3%)	9 (21·4%)
Number of people in household, Mean (SD)	2·9 (1·3)	3·4 (1·8)
Children in household (%)	8 (38·1%)	22 (52·4%)
Take public transit to work	12 (57·1%)	27 (64·3%)
Direct patient care	9 (42·9%)	15 (40·5%)
Influenza vaccination history		
2007–2008	12/21 (57·1%)	22/41 (53·6%)
2006–2007	14/19 (73·7%)	27/36 (75·0%)
2005–2006	14/17 (82·3%)	26/35 (74·3%)

* Compared with never or former smoker.

**Figure 2:**
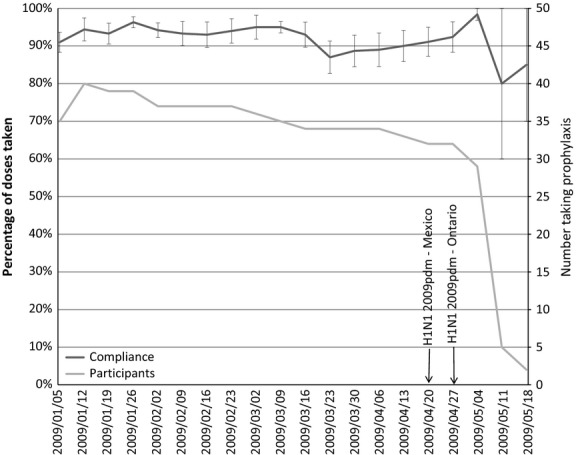
Number of participants taking antiviral prophylaxis and the percentage of doses taken per week, January – May 2009.

The 2008–2009 influenza season started during week 53 (28 December 2008) and the study continued until 30 May 2009 (week 21). The pandemic A/California/07/2009 (H1N1) strain started circulating in Ontario during week 17 (26 April 2009) and the first wave peaked during weeks 22–24 (31 May 2009 to 20 June 2009).^19^ Participants in the prophylaxis group started taking their medication on 5 January 2009, with 35/40 starting during the first week and the other 5 starting during the second week of January. Three individuals discontinued prophylaxis due to side effects: one in week 1 with nausea, vomiting and headache and two people in week 4, one with joint pain and one with increased nasal congestion. All three were vaccinated upon discontinuation. After 13 weeks (April 6th), the expected duration of the season and study, participants in the antiviral group were given the options of discontinuing prophylaxis and being vaccinated (*n* = 1, who was vaccinated April 7th but restarted prophylaxis April 29th due to the increased local pandemic influenza activity), discontinuing prophylaxis without vaccination (*n* = 4), continuing prophylaxis for an additional 4 weeks/the next planned study visit (*n* = 3) or continuing for an additional 5·5 weeks/end of study (*n* = 29). Medication was taken for a median of 121 days (range 6–130) with very good adherence. According to self-reports on weekly diaries, 35/40 participants took >90% of prescribed doses while using prophylaxis. Adherence was higher at the start of prophylaxis with an average of 94% of doses taken per week until March 16th but dropped to 91% from March 17th to May 11th (see Figure2).

Baseline levels of seroprotection to the strains included in the 2008–2009 seasonal influenza vaccine were similar in the two groups. As shown in Table2, the vaccine met U.S.A. Center for Biologics Evaluation and Research guideline criteria for both seroprotection and seroconversion for A/Uruguay/716/2007 (H3N2) but met neither criterion for A/Brisbane/59/2007 (H1N1) or B/Florida/4/2006. In seasonal vaccines produced for Canada, the A/Uruguay/716/2007 (H3N2) antigenic strain was used in place of the WHO-recommended A/Brisbane/10/2007 (H3N2) strain.^20^ Thus, while 86% seroconverted to A/Uruguay/716/2007 (H3N2) following vaccination, only 24% of participants seroconverted to A/Brisbane/10/2007 (H3N2), the strain that circulated in 2008–2009.

**Table 2:** Rates of seroprotection based on hemagglutination inhibition assays of serology collected at baseline, post-vaccine or medication start, mid-season, and end-of-season, Toronto, Ontario, 2008–2009

Group	Influenza strain	Baseline October 2008 per cent (95% CI)	Post-vaccine/medication start* per cent (95% CI)	Mid-season March 2009 per cent (95% CI)	End-of-season June 2009 per cent (95% CI)
Vaccine group (*n* = 21)	A/Brisbane/59/2007 (H1N1)**	28·6 (9, 48)	66·7 (46, 87)	40·0 (18, 61)	30·0 (10, 50)
	A/Uruguay/716/2007 (H3N2)**	9·5 (0, 22)	85·7 (71, 100)	65·0 (44, 86)	50·0 (28, 72)
	A/Brisbane/10/2007 (H3N2)	0	23·8 (6, 42)	10·0 (0, 23)	5·0 (0, 15)
	B/Florida/4/2006**	57·1 (36, 78)	71·4 (52, 91)	65·0 (44, 86)	70·0 (50, 90)
	B/Malaysia/2506/2004	38·1 (17, 59)	42·9 (22, 64)	30·0 (10, 50)	30·0 (10, 50)
Antiviral group (*n* = 40)	A/Brisbane/59/2007 (H1N1)**	17·5 (6, 29)	15·0 (4, 26)	17·5 (6, 29)	10·3 (1, 20)
	A/Uruguay/716/2007 (H3N2)**	15·0 (4, 26)	17·5 (6, 29)	20·0 (8, 32)	20·5 (8, 33)
	A/Brisbane/10/2007 (H3N2)	10·0 (1, 19)	12·5 (2, 23)	17·5 (6, 29)	15·4 (4, 27)
	B/Florida/4/2006**	62·5 (47, 77)	52·5 (37, 68)	55·0 (40, 70)	53·8 (38, 69)
	B/Malaysia/2506/2004	30·0 (16, 44)	27·5 (14, 41)	27·5 (14, 41)	28·2 (14, 42)

* Post-vaccine serology drawn 2–3 weeks following vaccination; Medication start (antiviral group) was January 5–15, 2009.

** Strains in the 2008–2009 trivalent inactivated influenza vaccine.

Seroprotection defined as HAI titres ≥1:40. Three participants in the antiviral arm were vaccinated between medication start and end-of-season (included); see Figure 1 for participants per blood draw.

### Incidence of influenza and acute respiratory illnesses

Four cases (6·2 per 100 person-seasons) of influenza infection were detected. Three cases were symptomatic and detected by NAAT while the fourth was detected by serology alone. Two cases occurred in participants from the vaccine arm: both tested positive to influenza A (H1N1, seasonal strain) but neither had a significant increase in HAI titres to tested strains. One participant randomized to antiviral prophylaxis, who reported taking 93% of prescribed doses, tested positive for influenza B by NAAT on 18 February 2009, but had no change in HAI titres. A second person taking antiviral prophylaxis did not report symptoms of an acute respiratory illness on questionnaires nor when questioned during the final study visit, but had a 16-fold increase in antibody titres to A/California/07/2009 (H1N1). This individual reported 100% compliance with the prophylaxis, stopped taking medication May 25th and had their final study visit (and blood draw) on July 15th. The period between discontinuing medication and the blood draw was during the peak of the first wave of the pandemic in Ontario^19^ making it possible that the participant was exposed after discontinuing zanamivir prophylaxis. This case was not used in the analyses for effectiveness.

As shown in Table3, 109 episodes of acute respiratory illness were by reported by 47 participants, with 0–9 episodes per participant. There was no difference in the number of episodes for people receiving prophylactic antiviral medication compared to those receiving seasonal influenza vaccine before (RR 0·78 [CI_95%_ 0·51, 1·18] *P* = 0·25) or following adjustment for week of illness (as shown in Table4). Results of unadjusted conditional logistic regression analysis matched on week of illness (Table4), determined that odds of reporting an illness were higher for older participants and those who provided direct patient care. The odds of reporting an acute respiratory illness also increased with recent exposure (past 7 days) to a patient, co-worker, spouse or child who had symptoms of a respiratory illness. Using a multivariable model to adjust for the impact of other factors, the odds of reporting an acute respiratory illness doubled following recent exposure to a patient or child who had symptoms but were 7 times higher following exposure to a spouse with symptoms of an acute respiratory illness.

**Table 3:** Acute respiratory illnesses reported by questionnaire and swabs submitted for testing; Toronto November 2008 to May 2009

	Vaccine group (*n* = 20)	Antiviral group (*n* = 40)	Total
Influenza (A or B)	2	1	3
Human coronavirus	8	16	24
Rhinovirus/Enterovirus	0	3	3
Respiratory syncytial virus	0	2	2
Negative for viruses	12	48	60
Illness without swab	8	9	17
Total (Attack rate)	30 (1·5%)	79 (2·0%)	109

**Table 4:** Unadjusted and adjusted odds ratios for acute respiratory illnesses, conditional on week of report, Toronto Canada, November 2008 to May 2009

	Odds ratio*	*P* value	95% CI	Adjusted odds ratio**	*P* value	95% CI
Study arm						
Vaccine	Ref			Ref		
Antiviral	1·33	0·16	0·89, 1·98	1·30	0·24	0·84, 2·01
Age (years)	1·02	0·044	1·00, 1·04	–		
Male	Ref			–		
Female	1·07	0·72	0·74, 1·54			
Never/former smoker	Ref			–		
Current	1·35	0·08	0·97, 1·90			
Does not use public transport	Ref			–		
Uses public transport	1·39	0·13	0·91, 2·12			
No direct patient care	Ref			–		
Direct patient care	1·82	0·003	1·23, 2·70			
Time-varying (weekly exposures)						
No exposure to ill patient	Ref			Ref		
Patient with ARI past week	2·20	0·009	1·22, 3·99	2·02	0·029	1·07, 3·81
No exposure to ill spouse	Ref			Ref		
Spouse with ARI past week	9·80	<0·001	3·81, 25·2	7·22	<0·001	2·71, 19·2
No exposure to ill adult	Ref			–		
Household adult with ARI	1·35	0·72	0·26, 7·01			
No exposure to ill child	Ref			Ref		
Household child with ARI	3·50	0·002	1·56, 7·87	2·37	0·034	1·07, 5·26
No exposure to ill co-worker	Ref			–		
Co-worker with ARI	2·06	0·044	1·02, 4·18			

ARI, acute respiratory illness (2 or more of: runny or stuffy nose, cough, sore throat, headache or fever); OR, odds ratio; Ref, Referent group.

* Conditional on week of report.

** Adjusted for other variables in model and conditional on week of report.

## Conclusions

Participants in this randomized controlled study were largely able and willing to use antiviral prophylaxis against influenza for an entire influenza season with 90% of participants taking at least 90% of scheduled doses for up to 18·5 weeks of prophylaxis. In this study, 7·5% of people discontinued the antiviral medication due to side effects. This is similar to an unblinded randomized controlled trial of adults taking oseltamivir or given influenza vaccine for the prevention of influenza infection. In that study, 10% of participants discontinued prophylaxis due to side effects over a full influenza season.^21^ In comparison, in three blinded, randomized, placebo-controlled trials only 1–2% of participants in each study arm discontinued zanamivir, oseltamivir or placebo prophylaxis due to side effects experienced during treatment.^6^–^8^

In adults, seasonal influenza vaccines prevent about 59% of laboratory-confirmed influenza illnesses compared with no vaccine.^5^ In randomized, placebo-controlled studies of antiviral prophylaxis, the protective efficacy of zanamivir and oseltamivir against laboratory-confirmed influenza ranged from 31 to 83% in adults and adolescents in studies that ranged in duration from 28 days to 6 weeks.^6^–^8^ Studies that directly compare vaccination and antiviral prophylaxis, including the current study and another pilot study conducted in our facility in the 2007–2008 season,^21^ highlight the fact that neither option is perfect, but that both provide greater protection than no intervention. Antiviral prophylaxis may provide protection for those at higher risk of contracting, transmitting, and/or suffering severe consequences of infection caused by influenza strains not included in the seasonal vaccines (e.g. during a pandemic or a year with significant mismatch between the vaccine and circulating strains).

In this study, the direct provision of patient care and exposure to a patient with symptoms of an acute respiratory infection in the previous week were both associated with higher odds of illness. This is consistent with other studies reporting that people working in healthcare institutions are at higher risk of acute respiratory illness than people working in other professions.^9^,^10^,^22^ Williams *et al*.^10^ report that people working in acute care hospitals in Germany had higher odds of reporting ARI during the 2006–2007 influenza season than people working for other employers after adjusting for age, sex and smoking status. In the current study, the risk of an acute respiratory illness was also higher for participants who were exposed to their spouse or child who was ill during the previous week. Exposure to an ill family member was reported as a risk factor for influenza for healthcare workers during the 2009 pandemic^23^, while living with^10^,^22^,^23^ or being otherwise exposed to children^9^ was a risk for respiratory illnesses in several other studies.

A limitation of this pilot study was the inability to blind participants to their treatment which may have biased both the reporting of respiratory symptoms and side effects of the antiviral medication. Secondly, participants provided exposure information after they reported ARI, which likely biased their recall of exposure to people who had symptoms of an acute respiratory illness. Also, since the 2009 pandemic strain of influenza A started to circulate towards the end of the planned study period, it likely increased long-term adherence to the prophylactic antiviral medication. In a previous study, it was found that adherence started to wane towards the end of the influenza season.^21^ As most of the study participants worked in an acute care facility and had regular internet access, it is likely that our study population was of a higher socioeconomic status than the general population which may limit the generalizability of the findings. Strengths of the study are that it was randomized and an overwhelming majority of participants consistently completed weekly diaries reducing recall bias of symptoms and exposures.

Neuraminidase inhibitors are effective in preventing influenza infections and adults can comply with a schedule of once daily doses for a full season, with a low percentage having to discontinue because of side effects. Exposure to a spouse, child or patient with symptoms of an acute respiratory illness within the past week are risk factors for respiratory illness in healthy adults exemplifying the need to follow guidelines to prevent the spread of respiratory infections, both at home and in the workplace.
